# Preferences for mechanical ventilation modes among intensivists in Türkiye: a nationwide point-prevalence study

**DOI:** 10.55730/1300-0144.6138

**Published:** 2026-01-24

**Authors:** Süleyman YILDIRIM, Nurhayat KILINÇ ÖZGÜN, Özcan ALPDOĞAN, Hüseyin UÇAR, İmren TAŞKIRAN, Adnan ATA, Özhan ÖZCAN, Kıvanç ÖNCÜ, Saba Mukaddes ACARBAY, Temel GÜNER, Pınar ÖZGÜN, Zerrin ÖZÇELİK, Ayşegül ÇINAROĞLU, Gizem KURADA, L. Serap AVLAĞI, Onur GÖKÇE, Özkul YILMAZ ÇOLAK, Fatma ÜLGER, Melda İŞEVİ, Özgür KILIÇ, Rahime AYDIN KAYALI, Metin YARICI, Berkay KÜÇÜK, Fatma YILDIRIM, Kamil GÖNDEREN, Bişar ERGÜN, Kutlay AYDIN, Kamuran ULUÇ, Sevda ONUK, Mine ALTINKAYA ÇAVUŞ, Yelda BALIK, Ahmet SARI, Hayriye CANKAR DAL, Şerife GÖKBULUT BEKTAŞ, Sema TURAN, Ferhan DEMİRER AYDEMİR, Murat GÜNEŞ, Canan GÜRSOY, Hüseyin Oğuz YILMAZ, Kamil İNCİ, Türkay AKBAŞ, Hamza GÜLTEKİN, Burcu ACAR ÇİNLETİ, Tuğçe MENGİ, Kaniye AYDIN, Ferhat ÇETİNKAYA, Selin EYÜPOĞLU, Elif KERİMOĞLU, Hüseyin ÖZKÖK, Selçuk YAYLACI, Havva KOCAYİĞİT, Burak KAYA, Mete ERDEMİR, Gürhan TAŞKIN, Kazım ROLLAS, Hüseyin ÖZKARAKAŞ, Mensure ÇAKIRGÖZ, Cenk KIRAKLI

**Affiliations:** 1 Intensive Care Unit, İzmir School of Medicine, University of Health Sciences, Dr. Suat Seren Chest Disease and Thoracic Surgery Training and Research Hospital, İzmir, Turkiye; 2 Intensive Care Unit, İzmir City Hospital, İzmir, Turkiye; 3 Intensive Care Unit, İzmir School of Medicine, University of Health Sciences, Tepecik Training and Research Hospital, İzmir, Turkiye; 4 Department of Intensive Care Unit, Kocaeli City Hospital, Kocaeli, Turkiye; 5 Intensive Care Unit, Sinop Atatürk State Hospital, Sinop, Turkiye; 6 Department of Anesthesia and Reanimation, Sinop Ataturk State Hospital, Sinop, Turkiye; 7 Intensive Care Unit, Sancaktepe Şehit Prof. Dr. İlhan Varank Education and Training Hospital, İstanbul, Turkiye; 8 Intensive Care Unit, Kastamonu Training and Research Hospital, Kastamonu, Turkiye; 9 Department of Anesthesiology and Reanimation, Kastamonu Training and Research Hospital, Kastamonu, Turkiye; 10 Department of Anesthesiology and Intensive Care, Prof. Dr. Cemil Taşcıoğlu State Hospital, İstanbul, Turkiye; 11 Intensive Care Unit, Eskişehir Yunus Emre State Hospital, Eskişehir, Turkiye; 12 Division of Intensive Care, Department of Anesthesiology and Reanimation, Faculty of Medicine, Ondokuz Mayıs University, Samsun, Turkiye; 13 Division of Intensive Care, Department of Internal Medicine, Faculty of Medicine, Ondokuz Mayıs University, Samsun, Turkiye; 14 Department of Critical Care, Ankara Etlik City Hospital, Ankara, Turkiye; 15 Pulmonary Intensive Care Unit, Department of Pulmonary Medicine, Ankara Etlik City Hospital, Ankara, Turkiye; 16 Department of Internal Medicine, Faculty of Medicine, İzmir Katip Çelebi University, Ataturk Training and Research Hospital, İzmir, Turkiye; 17 Department of Internal Medicine and Critical Care, Tekirdağ State Hospital, Tekirdağ, Turkiye; 18 Intensive Care Unit, Aydın State Hospital, Aydın, Turkiye; 19 Intensive Care Unit, Muş State Hospital, Muş, Turkiye; 20 Intensive Care Unit, Kayseri City Hospital, Kayseri, Turkiye; 21 Intensive Care Unit, Hamidiye Faculty of Medicine, University of Health Sciences, Haydarpaşa Numune Health Application and Research Center, İstanbul, Turkiye; 22 Department of Intensive Care, University of Health Sciences, Ankara Bilkent City Hospital, Ankara, Turkiye; 23 Intensive Care Unit, Çanakkale Mehmet Akif Ersoy State Hospital, Çanakkale, Turkiye; 24 Intensive Care Unit, Gümüşhane State Hospital, Gümüşhane, Turkiye; 25 Division of Intensive Care, Department of Anesthesiology and Reanimation, Faculty of Medicine, Muğla Sıtkı Koçman University, Muğla, Turkiye; 26 Intensive Care Unit, Muğla Training and Research Hospital, Muğla, Turkiye; 27 Division of Critical Care, Department of Internal Medicine, Faculty of Medicine, Gazi University, Ankara, Turkiye; 28 Division of Intensive Care, Department of Internal Medicine, School of Medicine, Düzce University, Düzce, Turkiye; 29 Department of Neurology, Faculty of Medicine, Dicle University, Diyarbakır, Turkiye; 30 Intensive Care Unit, Department of Neurology, Faculty of Medicine, İzmir Demokrasi University, İzmir, Turkiye; 31 Division of Medical Intensive Care Unit, Department of Internal Medicine, School of Medicine, Cukurova University, Adana, Turkiye; 32 Intensive Care Unit, Ordu State Hospital, Ordu, Turkiye; 33 Intensive Care Unit, Ordu University Training and Research Hospital, Ordu, Turkiye; 34 Intensive Care Unit, Sakarya University Training and Research Hospital, Sakarya, Turkiye; 35 Intensive Care Unit, Department of Anesthesiology and Reanimation, Sakarya University Training and Research Hospital, Sakarya, Turkiye; 36 Department of Intensive Care, Gülhane Training and Research Hospital, Ankara, Turkiye; 37 Intensive Care Unit, Department of Anesthesiology and Reanimation, İzmir School of Medicine, University of Health Sciences, Bozyaka Training and Research Hospital, İzmir, Turkiye

**Keywords:** Point prevalence study, mechanical ventilation, intensive care unit, synchronized intermittent mandatory ventilation, ventilation modes, weaning

## Abstract

**Background/aim:**

Invasive mechanical ventilation (IMV) is a fundamental intervention for patients with respiratory failure in intensive care units (ICUs). This nationwide, multicenter point-prevalence study aimed to describe current mechanical ventilation mode preferences (conventional, adaptive, and biphasic) in Turkish ICUs and to report associated clinical outcomes descriptively, without assessing causal relationships.

**Materials and methods:**

A nationwide, multicenter point-prevalence study was conducted on 17 April 2024 and included adult patients (≥18 years) who had been receiving IMV for more than 24 h. Data on patient demographics, ventilation mode distribution, ventilatory parameters, and descriptive clinical outcomes on day 28 (weaning status, tracheostomy, and mortality) were recorded without comparative outcome analysis.

**Results:**

A total of 426 patients were included. Conventional modes were used in 84.5% of patients, adaptive modes in 10.6%, and biphasic modes in 4.9%. Synchronized intermittent mandatory ventilation (SIMV) was the most commonly used conventional mode. The primary indication for IMV was acute respiratory failure (61%), with pneumonia being the leading cause. Among the 350 orotracheally intubated patients, 25.6% were in the weaning phase on the study day. A total of 59 (16.9%) patients were extubated, 150 (42.9%) underwent tracheostomy, and 64 (18.2%) remained intubated on day 28. Overall, 185 (43.4%) patients died during their ICU stay, 152 (35.7%) remained in the ICU, and 89 (20.9%) were successfully discharged from the ICU.

**Conclusion:**

Conventional ventilation modes, particularly SIMV, were more commonly used in Turkish intensive care units (ICUs), whereas adaptive modes were less frequently applied. These patterns may reflect factors such as clinician familiarity, institutional practices, and equipment availability rather than definitive preferences. Although the impact of ventilation modes on clinical outcomes was not comparatively evaluated in this study, the choice of ventilation mode may still influence patient outcomes. Therefore, further prospective and comparative studies are warranted to better elucidate this relationship.

## 1. Introduction

Invasive mechanical ventilation (IMV) is a life-saving intervention widely employed in critical care settings to manage patients with respiratory failure. Mechanical ventilators support gas exchange by delivering flow and pressure to maintain adequate oxygenation and ventilation. A lung-protective ventilation strategy—characterized by lower tidal volumes and limited plateau pressures—is recommended for critically ill patients, both with and without acute respiratory distress syndrome (ARDS) [1–4]. Clinicians adjust these parameters through various mechanical ventilation (MV) modes.

Since the introduction of positive-pressure ventilators, more than 200 MV modes have been described. These modes are broadly classified into three categories: conventional modes, in which the ventilator strictly adheres to operator-defined settings; adaptive modes, in which control settings are automatically adjusted; and biphasic modes, in which the baseline pressure alternates between two positive-pressure levels [5]. The modes of mechanical ventilation determine how a mechanical breath is initiated, maintained, and terminated. The selection of the appropriate ventilation mode is crucial, as it directly impacts patient outcomes, including comfort, oxygenation, and weaning from mechanical ventilation [6–9]. Despite the availability of various ventilation modes, the choice of ventilation mode is often influenced by several factors, including the patient’s clinical condition, physician experience, and institutional protocols.

In Türkiye, there are limited data on the preferences and practices surrounding MV modes in intensive care units (ICUs). Understanding preferences for different mechanical ventilation modes among healthcare providers is essential for optimizing patient care. These preferences may be guided by factors such as ease of use, perceived efficacy, and the ability to individualize treatment according to patient needs. Moreover, emerging technologies and ventilation modes continue to evolve, offering new opportunities and challenges in mechanical ventilation management.

This study aimed to provide a comprehensive snapshot of MV practices in Turkish ICUs, focusing on the prevalence and preferences of various MV modes. By analyzing patient demographics, clinical characteristics, and ventilatory parameters, we sought to identify factors influencing ventilation mode selection and their implications for patient outcomes. This work represents the first nationwide point-prevalence study of MV practices in Türkiye and offers critical insights to guide future practice and research.

## 2. Materials and methods

### 2.1. Study design and setting

This was a prospective, national, multicenter point-prevalence study conducted across 37 participating intensive care units (ICUs) in 20 cities across Türkiye on 17 April 2024. Participating centers included tertiary university hospitals, training and research hospitals, city hospitals, and state hospitals, reflecting a broad spectrum of routine intensive care practices. The study centers were geographically distributed across multiple regions of the country, including İzmir, İstanbul, Ankara, Kocaeli, Sinop, Kastamonu, Eskişehir, Samsun, Tekirdağ, Aydın, Muş, Kayseri, Çanakkale, Gümüşhane, Muğla, Düzce, Diyarbakır, Adana, Ordu, and Sakarya. Data collection was performed simultaneously on a predefined study day at all participating ICUs.

Data were collected from ICUs in both academic and nonacademic hospitals to ensure broad representation of clinical practices nationwide. Pediatric ICUs and postoperative recovery areas were excluded from the study. The study was conducted after approval by the Ethics Committee of İzmir School of Medicine, University of Health Sciences, Dr. Suat Seren Chest Disease and Thoracic Surgery Training and Research Hospital (Ethical approval no: 2023/64-64; date: 15 November 2023). Informed consent was obtained from the patients or their legally authorized representatives. This study was conducted in accordance with the Declaration of Helsinki (2013).

### 2.2. Study population

**Figure 1:**
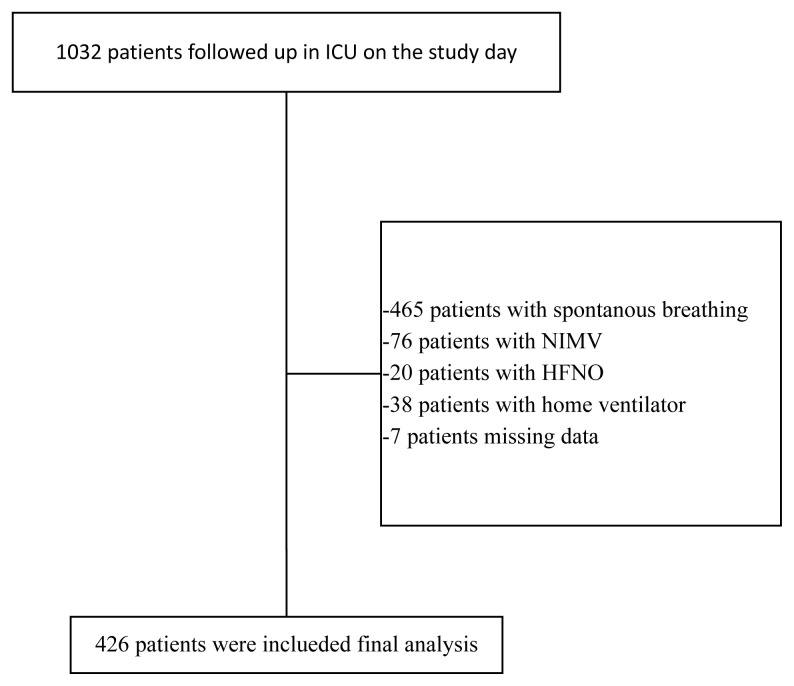
Flowchart of patient selection. This figure illustrates the inclusion and exclusion process of the study population on the designated study day. Adult patients (≥18 years) receiving invasive mechanical ventilation for more than 24 h were included. Patients receiving noninvasive ventilation, high-flow nasal oxygen therapy, or long-term home mechanical ventilation via tracheostomy were excluded.

We included adult patients (≥18 years) who had been receiving IMV for more than 24 h on the designated study day. Patients who underwent IMV for less than 24 h on the study day were not included. Patients who had undergone tracheostomy due to chronic respiratory failure and were receiving home mechanical ventilator were excluded from the study. Patients receiving noninvasive mechanical ventilation and those receiving high-flow nasal oxygen were also excluded. The flowchart of the study population selection is shown in Figure 1.

### 2.3. Data collection

Data were collected on a single day to capture a point-prevalence snapshot of current mechanical ventilation practices. Trained ICU personnel obtained information from electronic medical records and bedside charts. For each patient, demographic and clinical characteristics were collected, including age, sex, body weight, comorbidities, severity of illness (APACHE-II and SOFA scores on the study day), and the indication for IMV. The indication for IMV was categorized into four groups: acute respiratory failure, exacerbation of chronic obstructive pulmonary disease (COPD), neurologic disturbance, and neuromuscular dysfunction. Acute respiratory failure was further divided into the following subgroups: ARDS, acute pulmonary edema, pneumonia, aspiration, sepsis, postoperative causes, and trauma.

Ventilator settings were recorded, including the mode of mechanical ventilation, tidal volume, respiratory rate, positive end-expiratory pressure (PEEP), and fraction of inspired oxygen (FiO_2_). The blood gas and laboratory parameters closest to the time of data collection were also documented, including pH, PaO_2_, PaCO_2_, and lactate levels. We also recorded weaning from mechanical ventilation, tracheostomy status, and mortality on day 28. Patients were classified as being in the weaning phase based on clinical judgment at the participating centers.

### 2.4. Mechanical ventilation modes

The primary outcome was the mode of mechanical ventilation in use at the time of the study. Ventilation modes were categorized as follows:

- Conventional modes: continuous mandatory ventilation (CMV), synchronized intermittent mandatory ventilation (SIMV), assist-control ventilation (AC), and pressure support ventilation (PSV).
- Adaptive modes**:** adaptive support ventilation (ASV) and pressure-regulated volume control (PRVC).
- Biphasic modes**:** airway pressure release ventilation (APRV) and bilevel positive airway pressure.

We also documented ventilation modes for patients in the weaning phase, defined as the process of reducing ventilatory support to achieve extubation. Patients were classified as being in the weaning phase based on clinical judgment.

### 2.5. Statistical analysis

Descriptive statistics were used to summarize demographic characteristics, clinical parameters, ventilation settings, and outcomes. Continuous variables are presented as medians and interquartile ranges (IQR) because nonnormal distributions were identified using the Shapiro–Wilk test. Categorical variables are displayed as frequencies and percentages. Given the point-prevalence design of the study, clinical outcomes were recorded descriptively, and no causal or comparative analyses between ventilation modes and outcomes was intended or performed. All statistical analyses were conducted using SPSS (version 26; IBM Corp., Armonk, NY, USA), with a two-sided p-value <0.05 considered statistically significant.

## 3. Results

### 3.1. Patient demographics and clinical characteristics

A total of 426 adult patients receiving invasive mechanical ventilation (IMV) were included in this national point-prevalence study. The majority of the study population consisted of male patients (57.7%). Overall, the cohort was predominantly elderly (median age, 73 years) and was characterized by a high burden of comorbidities and significant illness severity (median APACHE-II score, 24). Detailed demographic characteristics and comorbidities are summarized in Table 1.

### 3.2. Indications for and types of mechanical ventilation

Acute respiratory failure was the most common indication for invasive IMV in 61.0% of patients, followed by neurologic disturbance (20.0%) and exacerbation of COPD (15.7%). Among patients with acute respiratory failure, pneumonia (38.0%) was the leading cause, followed by postoperative causes (10.3%) and aspiration (10.3%). A total of 36 (8.5%) patients underwent IMV due to acute respiratory distress syndrome (ARDS). Orotracheal intubation was used in 82.2% of cases, whereas 17.8% of patients had a tracheostomy.

**Figure 2:**
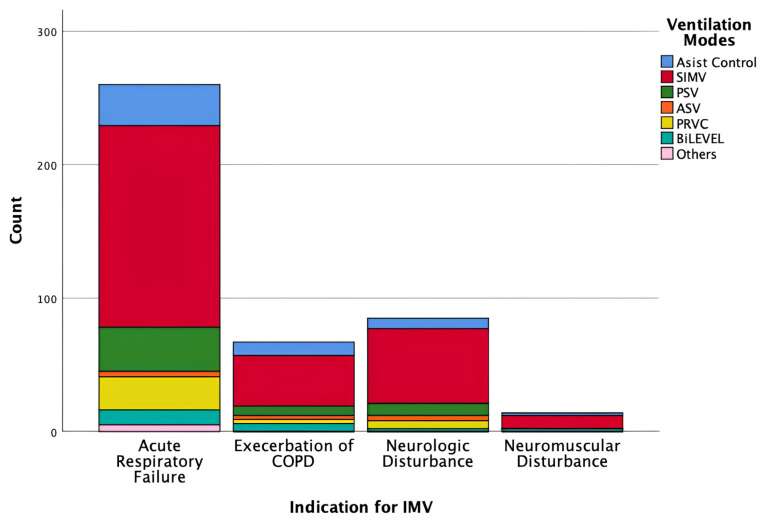
Ventilation mode preferences among all patients. This figure demonstrates the relative use of conventional, adaptive, and biphasic mechanical ventilation modes in the national point-prevalence cohort on the study day. Conventional modes include assist-control ventilation, synchronized intermittent mandatory ventilation (SIMV), and pressure support ventilation, whereas adaptive and biphasic modes are presented separately.

**Figure 3:**
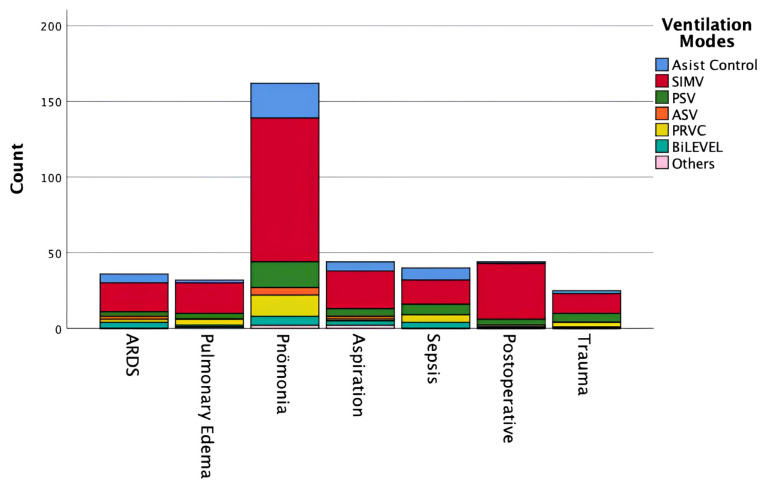
Ventilation mode preferences among patients with acute respiratory failure. This figure shows the distribution of mechanical ventilation modes among patients receiving invasive mechanical ventilation for acute respiratory failure on the study day, highlighting the predominance of conventional ventilation modes in this subgroup.

Conventional modes were the most frequently used ventilation modes, applied in 360 (84.5%) patients. Adaptive modes were used in 45 (10.6%) patients, and biphasic modes in 21 (4.9%) patients. Among conventional modes, synchronized intermittent mandatory ventilation (SIMV) was the most frequently used mode: V-SIMV + PS in 89 (20.9%) patients, P-SIMV + PS in 77 (18.1%) patients, P-SIMV in 56 (13.1%) patients, and V-SIMV in 33 (7.2%) patients. Other frequently used modes included PSV (11.5%) and PRVC (8.0%) (Table 2; Figure 2). Notably, SIMV was also the most frequently used mode across all subtypes of acute respiratory failure (Figure 3).

### 3.3. Mechanical ventilation parameters

Ventilatory settings reflected widespread application of lung-protective ventilation principles; the median tidal volume was 7.56 mL/kg, and the median plateau pressure (Pplat) was 19 cmH_2_O. The median respiratory rate was 15 breaths per minute, and the median FiO_2_ was 40%. The median positive end-expiratory pressure (PEEP) was 6 cmH_2_O. Oxygenation and ventilation parameters, as assessed by arterial blood gas analyses, showed a median pH of 7.45, with median values of PaO_2_ 81.5 mmHg, PaCO_2_ 41.0 mmHg, and SaO_2_ 96%. Detailed ventilatory parameters and laboratory values are summarized in Table 3.

### 3.4. Weaning and outcomes

Among the 350 orotracheally intubated patients, approximately one-quarter were considered to be in the weaning phase on the study day. SIMV (50.5%) and PSV (33.9%) were the most commonly used modes during this phase. A total of 59 (16.9%) patients were extubated, 150 (42.9%) underwent tracheostomy, and 64 (18.2%) remained intubated on day 28. The median time from intubation to extubation was 8 days (IQR, 4–16 days), whereas tracheostomy was performed after a median of 24 days (IQR, 19–28 days) from intubation.

Overall, 185 (43.4%) patients died during their ICU stay, 152 (35.7%) remained in the ICU, and 89 (20.9%) were successfully discharged from the ICU.

## 4. Discussion

This national point-prevalence study provides a descriptive overview of current mechanical ventilation practices in Turkish ICUs and demonstrates a predominance of conventional ventilation modes, particularly synchronized intermittent mandatory ventilation (SIMV). In contrast, adaptive ventilation modes were used less frequently. These findings reflect ventilation practices observed on the study day and should be interpreted as a snapshot of real-world practice rather than as evidence of superiority or preference for any specific ventilation strategy.

Globally, conventional ventilation modes remain prevalent, particularly assist-control (AC) ventilation, which gained popularity following its adoption in the ARDSnet clinical trial, especially among patients with ARDS [4]. Checkley et al. demonstrated a marked increase in A/C mode usage in patients with ARDS, rising from 61% to 82% between 1996 and 2005 [10]. The growing preference for A/C mode can be attributed to several factors. A/C ventilation offers greater control over patient ventilation by delivering a set tidal volume or pressure with each breath, regardless of whether the breath is initiated by the patient or the ventilator. This approach provides more consistent and reliable ventilation support, particularly for patients with compromised respiratory function who require continuous and stable assistance. Conversely, SIMV has lost popularity because it has been increasingly criticized for its association with patient–ventilator asynchrony, which may contribute to ventilator-induced lung injury and delayed weaning [11–13]. The use of SIMV decreased from 22% to 3% between 1996 and 2005 in patients with ARDS [10]. Similarly, in a recent study, SIMV use was reported in 5% of a mixed ICU population [14]. The use of SIMV tended to be higher in patients without ARDS. In the PRoVENT study, SIMV was the most frequently used ventilation mode in patients at risk of ARDS and in those not at risk [15]. SIMV was more frequently used in patients with lower disease severity, as well as in postoperative and trauma patients [16].

Despite accumulating international evidence questioning the role of SIMV, particularly during the weaning phase, SIMV remained the most frequently used ventilation mode in our cohort. This persistent use may be related to several real-world factors rather than evidence-based superiority. Clinician familiarity and long-standing training backgrounds, institutional routines shaped by historical practice patterns, and limitations related to ventilator availability or older device technology may all contribute to the continued preference for conventional modes such as SIMV. These factors highlight the complexity of ventilation mode selection in daily clinical practice.

Adaptive ventilation modes, including ASV and PRVC, represent an evolving trend in mechanical ventilation. By dynamically adjusting to patient’s changing respiratory needs, adaptive modes may reduce clinician workload and potentially improve patient comfort and outcomes. Adaptive modes may reduce the need for frequent clinician intervention by providing more dynamic and personalized ventilation, which may improve patient comfort and outcomes. A previous study showed that ASV shortened weaning time compared with PSV in patients with COPD [9]. In another study, PRVC improved blood gas parameters and weaning success compared with V-SIMV [17]. Recent international studies suggest a gradual shift from conventional ventilation modes toward innovative adaptive modes. ASV, one of the adaptive modes, was reported as the second most frequently used ventilation mode after A/C in a mixed ICU population [14]. Previous studies have shown that adaptive modes can improve outcomes in select patient populations, but further education and evidence may be required to support their broader adoption [18–20]. Despite their promise, adaptive modes were underutilized in our study. Barriers such as limited clinician experience, lack of education, and restricted availability of advanced ventilator technologies may contribute to their lower adoption. Expanding clinician training and ensuring access to newer ventilators could facilitate a broader transition toward adaptive ventilation. Conventional ventilation modes are available in nearly all mechanical ventilator brands, whereas adaptive ventilation modes are not available in many devices; therefore, the use of conventional modes may reflect necessity in certain settings rather than adherence to newer evidence or emerging technologies.

The choice of ventilation modes may have direct implications for patient outcomes, including oxygenation, weaning, and mortality. In our cohort, among the 350 orotracheally intubated patients, 109 (31.1%) were in the weaning phase on the study day, with SIMV being the most frequently used mode, followed by PSV (50.5% and 33.9%, respectively). By day 28, only 16.9% (59/350) of patients were extubated, whereas a considerably higher proportion, 42.9% (150/350), underwent tracheostomy. These extubation rates are lower and tracheostomy rates are higher than those reported in international cohorts; for example, in the WEAN SAFE study, 65% of patients receiving invasive mechanical ventilation for more than 2 days were successfully weaned by day 90, and tracheostomy was performed in only 19.6% of patients [21]. Practices of liberation from mechanical ventilation vary across ICUs and countries. Across regions, there was variation in the use of written directives to guide care, daily screening practices, spontaneous breathing trial techniques, ventilator modes, and the roles of clinicians involved in weaning [22]. Consistent with prior data from Türkiye, where successful weaning has been reported in only approximately one-quarter of invasively ventilated patients, our findings suggest a relatively low rate of liberation from mechanical ventilation [23].

In our study, the relatively low extubation rate and high tracheostomy rate are likely multifactorial and should be interpreted within the context of the study population and design. First, the patients included were of advanced age, which is known to be associated with prolonged mechanical ventilation and reduced weaning success. Second, the overall severity of illness in our cohort was high, reflecting a population with substantial organ dysfunction and complex clinical trajectories—factors that are commonly linked to delayed liberation from mechanical ventilation and increased tracheostomy requirements. Third, the point-prevalence design inherently captures patients at a single time point and may disproportionately represent individuals with longer ICU stays and prolonged ventilatory support, thereby overestimating tracheostomy rates while underestimating successful extubation. Accordingly, these findings should be interpreted descriptively rather than causally, and longitudinal studies are required to assess weaning outcomes and determinants of tracheostomy more accurately in this population.

Lung-protective ventilation is the primary strategy in patients undergoing mechanical ventilation. Our results showed that lung-protective ventilation was widely implemented in Turkish ICUs. Previous studies have reported poor adherence to lung-protective ventilation in Turkish ICUs. Kaya et al. reported that the median tidal volume was 9.3 mL/kg in patients without ARDS [24]. Increased awareness of lung-protective ventilation during the COVID-19 outbreak is considered a major contributing factor to this change [25,26].

There are several limitations to this study that should be acknowledged. First, the point-prevalence design inherently precludes causal inference regarding the relationship between ventilation mode selection and clinical outcomes. Second, the weaning phase was defined based on clinical judgment at participating centers rather than standardized criteria, which may have introduced intercenter variability. Third, although 28-day outcomes were reported, patients were not followed from a uniform baseline; therefore, these outcomes should be interpreted descriptively and with caution. In addition, ventilation mode selection was recorded at a single time point and may not reflect changes in ventilation strategies over the course of the ICU stay. Ventilation modes may be adjusted in response to evolving clinical status, sedation level, or progression through the weaning process—factors that could not be captured within the present study design.

In conclusion, this nationwide point-prevalence study provides valuable insights into mechanical ventilation practices in Turkish ICUs, highlighting a strong preference for conventional ventilation modes, particularly SIMV. Despite growing evidence supporting adaptive ventilation modes, their adoption remains limited, potentially due to clinician familiarity, equipment availability, and institutional protocols. Encouragingly, lung-protective ventilation strategies were widely observed, reflecting improved adherence to evidence-based practices. However, the relatively low extubation rates observed underscore the need to optimize weaning practices and evaluate the impact of ventilation mode selection on patient outcomes. Future prospective and longitudinal studies are warranted to better evaluate the impact of mechanical ventilation mode selection on clinical outcomes and weaning success.

## Figures and Tables

**Table 1:** Demographic characteristics of the patients.

	All patients (n = 426)
**Age, median (IQR), years**	73.0 (63.0–81.0)
**Sex, male, n (%)**	246 (57.7)
**Height, median (IQR), cm**	170 (160–175)
**Body weight, median (IQR), kg**	75.0 (65.0–85.0)
**Ideal body weight, median (IQR), kg**	66.0 (52.4–70.6)
**Comorbidities, n (%)**	
**Hypertension**	259 (60.8)
**Diabetes mellitus**	145 (34.0)
**Coronary artery disease**	136 (31.9)
**Chronic heart failure**	109 (25.6)
**COPD**	107 (25.1)
**Alzheimer/dementia**	86 (20.2)
**Solid organ malignancy**	70 (16.4)
**Hematologic malignancy**	13 (3.1)
**APACHE-2 Score, median (IQR)**	24 (19–29)
**SOFA score on study day, median (IQR)**	7 (5–10)

APACHE II: acute physiology and chronic health evaluation; COPD: chronic obstructive pulmonary disease; IQR: interquartile range; SOFA: sequential organ failure assessment.

**Table 2:** Clinical characteristics and modes of mechanical ventilation.

	All Patients (n = 426)
**Indication for IMV, n (%)**	
**Acute respiratory failure**	260 (61.0)
**Exacerbation of COPD**	67 (15.7)
**Neurologic disturbance**	85 (20.0)
**Neuromuscular dysfunction**	14 (3.3)
**Type of acute respiratory failure, n (%)**	
**Acute respiratory distress syndrome**	36 (8.5)
**Pulmonary edema**	32 (7.5)
**Pneumonia**	162 (38.0)
**Aspiration**	44 (10.3)
**Sepsis**	40 (9.4)
**Postoperative causes**	44 (10.3)
**Trauma**	25 (5.9)
**Other causes**	43 (10.1)
**Airway type, n (%)**	
**Orotracheal intubation**	350 (82.2)
**Tracheostomy**	76 (17.8)
**Mechanic ventilatory brand, n (%)**	
**Biovent**	107 (25.1)
**GE**	137 (32.2)
**Maquet**	71 (16.7)
**Hamilton**	49 (11.5)
**Drager**	20 (4.7)
**Puritan Bennett**	14 (3.3)
**Others**	28 (6.6)
**Ventilation mode, n (%)**	
**Conventional modes**	360 (84.5)
**V-CMV**	3 (0.7)
**P-CMV**	7 (1.6)
**V-AC**	23 (5.4)
**P-AC**	18 (4.2)
**V-SIMV**	33 (7.2)
**P-SIMV**	56 (13.1)
**V-SIMV+PS**	89 (20.9)
**P-SIMV+PS**	77 (18.1)
**PSV**	49 (11.5)
**Others**	5 (1.1)
**Adaptive modes**	45 (10.6)
**ASV**	11 (2.6)
**PRVC**	34 (8.0)
**Biphasic ventilation modes**	21 (4.9)
**BiLEVEL**	20 (4.7)
**APRV**	1 (0.2)
**Duration of IMV on the study day, median (IQR), days**	10 (3–16)
Weaning phase*, **n (%)**	
**In weaning phase**	90 (25.7)
**Not in weaning phase**	260 (74.3)
Weaning from mechanical ventilation*, **n (%)**	
**Extubation**	59 (16.9)
**Tracheostomy**	150 (42.9)
**Still intubated**	64 (18.2)
**Death**	77 (22.0)
**Time to extubation, median (IQR), days**	8 (4–16)
**Time to tracheostomy, median (IQR), days**	24 (19–28)
ICU disposition**, **n (%)**	
**ICU disposition**	89 (20.9)
**Still in ICU**	152 (35.7)
**Death**	185 (43.4)

AC: assist-control ventilation; APRV: airway pressure release ventilation; ASV: adaptive support ventilation; CMV: continuous mandatory ventilation; COPD: chronic obstructive pulmonary disease; IMV: invasive mechanical ventilation; IQR: interquartile range; PRVC: pressure-regulated volume control; PSV: pressure support ventilation; SIMV: synchronized intermittent mandatory ventilation.

* Among 350 orotracheally intubated patients.

** Among 426 patients who received invasive mechanical ventilation.

**Table 3:** Mechanical ventilation parameters.

	All patients (n = 426)
**Tidal volume, median (IQR), mL**	475 (430–500)
**Tidal volume, median (IQR), mL/kg**	7.56 (6.66–8.77)
**Respiratory rate, median (IQR)**	15 (12–18)
**FiO** **_2,_** ** median (IQR), %**	40 (35–50)
**PEEP, median (IQR), cmH** **_2_** **O**	6 (5–7)
**Ppeak, median (IQR), cmH** **_2_** **O**	27 (23–32)
**Pplat, median (IQR), cmH** **_2_** **O**	19 (16–24)
**Pmean, median (IQR), cmH** **_2_** **O**	21 (16–26)
**pH, median (IQR)**	7.45 (7.38–7.49)
**PaO** **_2_** **, median (IQR), mmHg**	81.5 (69.0–105.0)
**PaCO** **_2_** **, median (IQR), mmHg**	41.0 (35.8–47.2)
**SaO** **_2_** **, median (IQR), %**	96 (93–98)
**HCO** **_3_** **, median (IQR), mmol/L**	27.0 (23.7–30.2)
**Lactate, median (IQR), mmol/L**	1.7 (1.2–2.3)

IQR: interquartile range; PEEP: positive end-expiratory pressure.
